# A Recombinant Human Anti-Platelet scFv Antibody Produced in *Pichia pastoris* for Atheroma Targeting

**DOI:** 10.1371/journal.pone.0170305

**Published:** 2017-01-26

**Authors:** Amelie Vallet-Courbin, Mélusine Larivière, Agnès Hocquellet, Audrey Hemadou, Sarjapura-Nagaraja Parimala, Jeanny Laroche-Traineau, Xavier Santarelli, Gisèle Clofent-Sanchez, Marie-Josée Jacobin-Valat, Abdelmajid Noubhani

**Affiliations:** 1 UMR5248, CBMN, Bordeaux-INP, Pessac, France; 2 Centre de Résonance Magnétique de Systèmes Biologiques, Centre Nationale de Recherche Scientifique et Université de Bordeaux, Bordeaux, France; Institut d'Investigacions Biomediques de Barcelona, SPAIN

## Abstract

Cells of the innate and adaptive immune system are key factors in the progression of atherosclerotic plaque, leading to plaque instability and rupture, potentially resulting in acute atherothrombotic events such as coronary artery disease, cerebrovascular disease and peripheral arterial disease. Here, we describe the cloning, expression, purification, and immunoreactivity assessment of a recombinant single-chain variable fragment (scFv) derived from a human anti-αIIbβ3 antibody (HuAb) selected to target atheromatous lesions for the presence of platelets. Indeed, platelets within atheroma plaques have been shown to play a role in inflammation, in platelet-leucocyte aggregates and in thrombi formation and might thus be considered relevant biomarkers of atherosclerotic progression. The DNA sequence that encodes the anti-αIIbβ3 TEG4 scFv previously obtained from a phage-display selection on activated platelets, was inserted into the eukaryote vector (pPICZαA) in fusion with a tag sequence encoding 2 cysteines useable for specific probes grafting experiments. The recombinant protein was expressed at high yields in *Pichia pastoris* (30 mg/L culture). The advantage of *P*. *pastoris* as an expression system is the production and secretion of recombinant proteins in the supernatant, ruling out the difficulties encountered when scFv are produced in the cytoplasm of bacteria (low yield, low solubility and reduced affinity). The improved conditions allowed for the recovery of highly purified and biologically active scFv fragments ready to be grafted in a site-directed way to nanoparticles for the imaging of atherosclerotic plaques involving inflammatory processes and thus at high risk of instability.

## Introduction

Atherosclerosis is an inflammatory disease associated with the formation of unstable thrombosis-prone atheroma plaques made of large lipid cores, thin fibrous cap and inflammatory cell infiltrates within the walls of arteries.[1] Atherosclerotic plaque rupture is the mechanistic cause of about 75% of all sudden and often fatal heart attacks.[2] As the risk of plaque rupture is more related to the plaque contents than to the plaque size, molecular imaging modalities have risen as a new imperative. Current studies tend towards the development of non-invasive targeted methods to assess the cellular components that underlie the risk of rupture.[3,4] Molecular imaging requires highly sensitive and specific probes made of a signal detection compound and an affinity ligand for targeting. The affinity ligand should be able to recognize molecules and cells over-expressed during the course of atherogenesis. Inflammation is a well-recognized pathophysiological process involving both innate and adaptive immune cells.[5] Recruitment of monocytes in the vascular wall and macrophage differentiation and proliferation represent a hallmark in the pathology of atherosclerotic lesions.[6] They contribute to the processes that underlie atherogenesis such as lipid accumulation, secretion of pro-inflammatory cytokines, extracellular matrix remodelling. Moreover, the observation of activation and oligoclonal expansion of T cells has suggested the presence of inciting antigens (Ags) that sustain T cell recruitment within coronary lesions.[7] B cells also play a pro or anti-atherogenic role depending on the subtypes (B1(a) or B2), and in atherosclerosis they accumulate both in the atherosclerotic intima and associated adventitia.[8–10] More recently, platelets have come to the forefront as partners of macrophages, T cells and B cells in inflammation and immune responses. They are now recognized as key players in innate and adaptive immune responses [11,12] and notably shown to modulate the T-effector/T-regulator balance via the CD40 ligand.[13,14] Platelet-derived CD40 ligand has also been reported to support B-cell differentiation and immunoglobulin class switching in mice.[15] Several cytokines released by activated platelets have been demonstrated to modulate monocyte and macrophage function.[16] Moreover platelet—leukocyte interactions largely contribute to OxLDL uptake and foam cell formation.[17] A recent study has underlined the presence of platelets not only in thrombi and intraplaque hemorrhage but also in atheroma burden, around necrotic areas and neovessels, shedding light on the rationale for targeting platelets within atherosclerotic lesions.[18]

Today, antibodies are used for several applications in research, diagnostics, and therapy.[19] Technology improvements are focused on several approaches to manufacturing recombinant human antibodies.[20] Moreover, *in vitro* selection technologies such as antibody phage display or ribosomal display have accelerated the generation of these recombinant human antibodies.[21–23]

To develop a novel non-invasive targeting approach for atheroma, our team previously selected, using *in vitro* phage display biotechnology on activated platelets, a phage-scFv fully human antibody (HuAb) specific to the αIIbβ3 integrin, which is an integrin only expressed on platelets and not on other immune cells.[24] This human antibody was further processed as a whole human IgG_4_ molecule in baculovirus system.[18] We proved the maintenance of the bioactivity after grafting onto superparamagnetic nanoparticles dedicated to MRI imaging. However, the chemical functionalization was hard to proceed, time-consuming and we did not succeed in grafting more than one HuAb onto each nanoparticle [18]. To overcome these drawbacks and obtain a better conjugation ratio, another type of protein engineering has been applied to reduce the probe size. A scFv protein composed of the heavy (VH) and light (VL) chains of an antibody linked with a flexible peptide, has been constructed by recombinant DNA technology. The diameter of scFv fragments (5 nm), one-fifth the size of whole IgG, is more suitable for functionalizing relatively small nanoparticles. Compared to much larger forms of antibodies such as Fab, Fab’2 and IgG, scFv have lower retention times in non-target tissues and exhibit more rapid blood clearance and better penetration into targeted lesions.[25] Moreover, this recombinant form can be generated with tags for purification and site-specific attachment via engineered thiols to avoid loss of bioactivity. There are a variety of recombinant production systems for the generation of scFvs ranging from bacteria, filamentous fungi, insect cell lines to transgenic plants.[20,26] More specifically, studies reported the production of an anti-platelets scFv antibody in transient mammalian cells system (e.g, freestyle HEK 293F cells).[26] However, transient antibody production appears more suitable for small-scale production in antibody screening. Here, TEG4 HuAb needs to be stably expressed as scFv fragments in a quantity so that purification, characterization and further grafting of isolated recombinant fragments could be readily accomplished. Hence, TEG4 HuAb was processed in *Pichia pastoris*. The choice of production in yeast cells was guided by previous experiments where biological activity could only be recovered in the cytoplasm of cells when using *E*. *coli* as a host expression system. Indeed, despite improvements in fedbatch fermentation production, a proportion of inactive scFv remained in the total purified population.[27] From this point of view, the *Pichia pastoris* expression system is an attractive way due to its ability to secrete a large amount of recombinant protein in the supernatant, thereby facilitating the purification steps.

The aim of the study was thus to produce highly purified and biologically active scFv fragments in a suitable yeast cell expression modality. The biochemical characteristics of the anti-αIIbβ3 scFv were evaluated by ELISA, affinity binding analyses, flow cytometry against platelets and immunohistochemistry (IHC) on atheroma plaques from animal models and human coronary sections. These scFv fragments are ready to be grafted in a site-directed way to nanoparticles for the imaging of atherosclerotic plaques where inflammatory and immune processes increase the risk of instability.

## Materials and Methods

### Materials

Large-scale expression of TEG4-2c scFv in *Pichia pastoris* was carried out in BIOSTAT Bplus laboratory fermentor 5L (Sartorius-Stedim Biotech, Germany). Data and set points were monitored with MFCS-Win. Growth media were purchased from Becton Dickinson (Le Pont de Claix, France).

Immobilized Metal Affinity Chromatography (IMAC) was carried out on HisTrap^™^ excel column (id 5 mL, GE Healthcare, Sweden) containing resin charged with nickel ions. Buffers for chromatographic runs, and reagents were prepared using chemicals of analytical grade from Sigma-Aldrich (Saint Quentin Fallavier, France).

Chromatographic experiments were performed using ÄKTA pilot workstation (GE Healthcare) and were monitored with Unicorn 5.1 software. Protein detection was monitored at 215 and 280 nm.

Spectrophotometric measurements of samples drawn from the fermentor and BCA protein assay measurements were carried out using SAFAS UVmc2 double-beam UV-visible spectrophotometer (Société Anonyme de Fabrication d’Appareillages Scientifiques, Monaco). Flow cytometry experiments were performed on a FACSCanto I cytometer from BD Biosciences (Le Pont de Claix, France) and monitored using DIVA software.

The absorbance of ELISA assays was read at 405 nm using a CHAMELEON microplate reader from ScienceTec (Les Ulis, France).

Surface Plasmon Resonance (SPR) experiments on purified αIIbβ3 integrin (Kordia Life Sciences, Leiden, The Netherlands) were carried out using the BIAcoreTM 3000 (GE Healthcare Europe GmbH, Velizy-Villacoublay, France) equipped with research-grade CM5 sensor chips. Bio-Layer Interferometry (BLI) experiments on lyophilized platelets (Helena Biosciences Europe, Queensway S, UK) were performed using an Octet instrument (Octet Red96 Pall Life Sciences, Saint-Germain-en-Laye, France) and HIS2 (anti-penta Histidine Ab) sensors.

### Methods

#### 1-Vectors and Strains

The pHOG21 vector, kindly provided by M. Little (Affimed Therapeutics, Lademburg, Germany), pCR4-TOPO and pPICZαA (Invitrogen, Carlsbad, USA) plasmids were used for preparation of DNA constructs. Synthetic oligonucleotides were purchased at Eurogentec (Liege, Belgium). Cloning step was carried out with *Escherichia coli* strain JM109 (NewEnglands Biolabs, Ipswich, MA USA). *Pichia pastoris* strain X33 used for production of recombinant TEG4-2c scFv was obtained from Invitrogen (Carlsbad, CA, USA).

#### 2-Construction of an expression vector for TEG4-2c scFv

**Fig 1:**
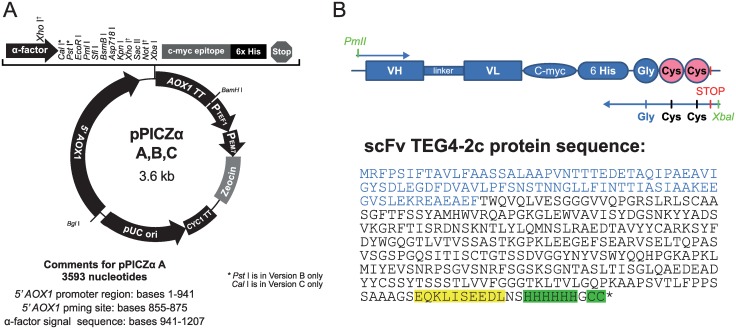
pPICZαA expression vector according to the EasySelect *Pichia* Expression Kit Manual (Invitrogen) and a schematic representation of TEG4-2c scFv with the protein sequence. **(A)**: All the featured restriction sites are unique. 5´ AOX1: promoter region of AOX1; TT AOX1: transcription termination of AOX1; PTEF1: promoter of TEF1; PEM7: promoter of EM7; Zeocin resistance: Sh ble ORF; CYC1 TT: transcription termination of CYC1. **(B)**: The TEG4-2c scFv coding sequence was cloned between *Pml*I and *Xba*I sites and the protein sequence of recombinant tag-scFv including the 6His-tag and the 2 cysteine are highlighted in green. The α-factor signal sequence is represented in blue and the C-myc tag is highlighted in yellow.

The TEG4 scFv DNA fragment provided in the pHOG21 vector previously used for the TEG4 scFv expression in XL1-Blue *E*. *coli* [16] was used as template to generate a new scFv format containing 2 cysteines into the C-terminal end. The coding sequence was PCR-amplified with *Pfu* Turbo polymerase (Stratagene). The primers (PICTE: 5’-TATCACGTGGCAGGTGCAGCTGGTGG-3’ and PIC2C: 5’-TCTAGATTAGCAGCACCCGTGATGGTGATGGTG-3’) were used to introduce the 6HisTag- Gly-Cyc-Cys-Stop amino acid sequence. Thirty amplification cycles were performed (30s at 95°C, 45s at 48°C and 1min at 72°C) followed by a final extension of 10 min at 72°C. The PCR product was purified, ligated into pCR4-TOPO plasmid and verified by DNA sequencing (Millegen Technology—France). The resultant plasmid was digested by *Pml*I and *Xba*I to excise TEG4-6His-Gly-Cys-Cys DNA fragment and ligated into the expression vector pPICZαA (Invitrogen, Carlsbad, USA) containing the Zeocin resistance gene for selection and the AOX1 promoter. The new construction of TEG4-2c scFv is shown in Fig 1.

The resultant plasmid was linearized by *Pme*I and transformed into competent *P*. *pastoris* X-33 cells by electroporation using Gene Pulser II (Bio-Rad, Hercules, CA, USA). Transformant cells were grown on YPDS-Zeocin 150 μg/mL-agar plates and screened again later for their ability to grow on Zeocin (Invitrogen, Carlsbad, USA) up to concentrations of 2 mg/ml. Selected clones were tested for the presence of TEG4-2c scFv coding sequence in their genome by colony PCR analysis using the PICTE and PIC2C primers, amplification cycles were preceded by a heating step 3 min at 95°C.

#### 3-Expression and purification of TEG4-2c scFv

Transformed *P*. *pastoris* cells that exhibited high resistance to Zeocin (up to 2 mg/mL plates) were grown in shake flasks containing 100 ml of buffered glycerol complex medium (BMGY, 1% yeast extract, 2% peptone, 100 mM potassium phosphate buffer at pH 6.0, 13.4g/L YNB, 4x10^-4^ g/L biotin, 10 g/L glycerol and 150 μg/mL Zeocin) until an optical density of 20 was reached. The culture was inoculated in a 5L bioreactor at 0.2 OD units. The bioreactor conditions were optimized by modification of Narasimhan, J *et al*.[28] The temperature and pH were maintained at 30°C and 6 respectively, and dissolved oxygen levels were maintained at 20% saturation by regulating aeration and agitation in a cascading system. After complete consumption of glycerol in the medium (20 to 24 hours), a methanol fed-batch phase was initiated by adding methanol every 12 h to a final concentration of 0.6%; this phase promotes the induction of scFv production and secretion in the medium. The methanol feed frequency was modified to every 6 h during the 4 latest days of production. Samples were drawn every 24 h to determine the yeast growth profile and scFv production. After 120 h of induction, the culture was harvested and the supernatant frozen at -80°C pending purification steps. Prior to purification, the pH of the broth was adjusted to 7.4 and filtered through a 0.45 μm cellulose acetate membrane.

The expressed recombinant TEG4-2c scFv was purified using IMAC. The HisTrap Excel resin was equilibrated with 50 mM Tris-HCl pH 7.5, 500 mM NaCl (buffer A at a flow rate of 3 mL/min). Typically, 450 to 800 mL *P*. *pastoris* expression broth supernatant containing the scFv was directly injected into the column. The column was then washed with buffer A until absorbance at 280nm reached the baseline. The elution was carried out into two steps using 5% and 30% buffer B (50 mM Tris-HCl pH 7.5, 500 mM NaCl, 500 mM imidazole) corresponding respectively to 25 mM and 150 mM imidazole. The elution fraction was dialyzed against PBS (NaCl 135 mM, KCl 2.5 mM, Na_2_HPO_4_ 10 mM, KH_2_PO_4_ 1.5 mM pH 7.4) buffer.

#### 4-Protein assay, SDS-PAGE and Dot-blot analysis

Protein quantification of the chromatographic fractions was performed using bicinchoninic acid protein micro-assay (BCA kit, Sigma). Twenty-five microliters samples were incubated with 200 μL of BCA working reagent and plate was incubated at 37°C for 30 min. The absorbance was measured at 562 nm.

Fractions obtained from chromatographic experiments were analyzed by SDS-PAGE under reducing conditions over 12% polyacrylamide gels. Supernatant and flow through fractions were 5x concentrated by TCA precipitation. Precision plus protein prestained standards (BIO-RAD) were used as molecular weight ladder.

To analyse the efficiency of the yeast cells *P*. *pastoris* to produce the scFv fragments into the broth medium, 50 μL samples from day 1 to day 5 were blotted on a nitrocellulose membrane using Bio-Dot Microfiltration Instrument (BIO-RAD). The membrane was blocked with a blocking buffer (TBS Tween with 3% milk powder) for 2 hours. The membrane was again washed twice with TBS-tween, and then incubated with primary antibody (Anti 6His, SIGMA) at 1:1500 dilution overnight at 4°C. Membranes were then washed and probed with a secondary antibody (anti-mouse IgG-HRP Cell Signaling Technology) at 1:5000 dilution.

Colorimetric analysis was performed using Opti4CN (BIO-RAD) kit by gently shaking until color develops.

#### 5-Preparation of Platelet-Rich-Plasma (PRP) and washed platelets

Platelet-rich plasma (PRP) and washed platelets were prepared from the blood of voluntary healthy donors.

PRP was obtained from venous blood anticoagulated with sodium citrate (0.38%, w/v) after centrifugation at 120 *g* for 10 min at room temperature.[29]

For the preparation of washed platelets, venous blood was anticoagulated with citric acid-citrate-dextrose NIH formula A (ACD-A) (1 vol of anticoagulant: 6 vol of blood). After centrifugation at 120 *g* for 10 min, the PRP was collected and mixed with ACD-A (1 vol: 9 vol, PRP) plus 100 nM prostaglandin E_1_ and 0.05 U/mL Apyrase ^Grade7^,[30] Platelets were sedimented by centrifugation at 1100 *g* for 15 min, washed and adjusted at 10^8^/mL in a modified Tyrode’s buffer (137 mM NaCl, 2.7 mM KCl, 12 mM NaHCO_3_, 0.3 mM NaH_2_PO_4_, 1 mM MgCl_2_, 5.5 mM glucose, 5 mM HEPES, 0.1% (wt/vol) bovine serum albumin (BSA), pH 7.4.

#### 6-Analysis of TEG4-2c scFv reactivity by Flow cytometry

**Analysis on washed platelets**

For the analysis of TEG4-2c scFv reactivity on platelets, **w**ashed platelets were activated with 0.5 U/ml human α-thrombin (Fibrindex, Ortho-Diagnostics, Raritan, NJ). Samples of both activated and non-activated platelets were fixed for 30 min with an equal volume of paraformaldehyde (PFA) 2% and then incubated with diluted TEG4-2c scFv.

Aliquots of 10^8^ PFA-fixed-washed-platelets/mL (10 μL) [27] non-activated (NA-PL) or activated with thrombin (A-PL) were incubated with 25 μg/mL of TEG4-2c scFv human antibody or PAC-1 commercial IgM antibody (BD Biosciences) [31] targeting the activated αIIbβ3 integrin overnight at 4°C. After two washes in PBS, a 30 min incubation with secondary Alexa Fluor 488 anti-6His or goat anti-mouse IgM antibodies (1:20) was performed for detection of respectively, the TEG4-2c scFv and the murine PAC-1 antibody. Reactions in the absence of antibodies were used as negative controls.

**Analysis on PRP samples**

Aliquots of 4 μL of PRP are used in each experiment. Some aliquots are activated with 50 μM thrombin receptor activating peptide (TRAP6; Sigma Aldrich, Saint-Quentin-Fallavier, France). Activated (+TRAP) and non-activated PRP aliquots (-TRAP) were then incubated with diluted TEG4-2c scFv (25 μg/ml) 15 min before adding secondary Alexa Fluor 488 anti-6His (both 1:20) antibody for another 15 min incubation. Reactions in the absence of antibodies were used as negative controls.

Cells were then resuspended in PBS before analysis on the FACSCanto I cytometer. The forward and wide-angle light scattering and fluorescence intensity from 10,000 platelets were collected using a logarithmic gain.

#### 7-Analysis of TEG4-2c scFv reactivity by ELISA

A 96-well flat-bottom polystyrene microtiter plate (Costar, Corning, NY) was coated with washed thrombin-activated (A-PL) or non-activated platelets (NA-PL) at 10^7^ platelets/well or BSA protein 20 μg/mL (Sigma-Aldrich) in 50 mM carbonate-bicarbonate buffer (pH 9.6) overnight at 4°C. The plate was washed three times with PBS (pH 7.4) containing 0.05% Tween 20 (200 μL/well) and blocked with 5% skimmed milk in PBS for 2 h at 37°C. TEG4-2c scFv fragments purified by IMAC or full-length mouse IgG antibody (AP2 antibody targeting αIIbβ3 integrin) [32] were tested on A-PL and NA-PL. After incubation for 2 h at 20°C, the plate was washed and incubated with an anti-6His IgG antibody (GE Healthcare) for 1 h at 37°C to detect scFv fragments or with PBS for full-length mouse antibodies. After washings, 100 μL of a 1:1000 dilution of horseradish peroxidase (HRP)-conjugated anti-mouse IgG (Immunotech, Marseille, France) was added and incubated for 90 min at room temperature. Color was developed with 100 μL of 2;2’-Azino-bis(3-ethylbenzothiazoline-6-sulfonic acid) (ABTS) (Sigma-Aldrich, Saint-Quentin, Fallavier, France) and the absorbance was read at 405 nm using a CHAMELEON microplate reader.

#### 8-Preparation of atheromatous and healthy aorta proteins

All animal experiments were performed in conformity with the Guide for the Care and Use of Laboratory Animals (NIH Publication No. 85–23, revised 1996) and were accredited by the local ethical committee.

Adult male New Zealand rabbits (NZW), weighting from 2.5 kg to 3.0 kg, were obtained from Charles Rivers Laboratories (St Germain sur l’Arbresle, France).

In order to mimic atherosclerosis that develop in humans, rabbits were fed a fat atherogenic diet including 0.3% cholesterol for 8 months and were subjected to surgeries to allow the formation of complex plaques with intramural thrombi. A first surgery was performed to remove endothelial cells from the thoracic until the abdominal aorta using a Fogarty catheter (Fogarty 4F; Edwards Lifesciences). The second surgery consists in an angioplasty using an expandable latex balloon (Maxxum, Boston Scientific; 20 mm long, diameter of 4.5 mm) under radioscopic guidance from the region of renal arteries to iliac bifurcation. Surgeries were performed under anesthesia by intramuscular administration of 20 mg/kg ketamine and 2 mg/kg xylazin. Anaesthesia was maintained with isoflurane gas (0.25% to 0.35%). As a preventive anti-thrombotic treatment, 1000 μI of heparine (Héparine Choay, Sanofi Synthélabo) was infused. After surgery 100 mg aspirine (Aspégic injectable, Sanofi Synthélabo) was administered as analgesia. Aortas from untreated rabbits and balloon-injured aortas from hypercholesterolemic rabbits were extracted from the aortic arch to the iliac bifurcation, washed and fractioned in order to solubilize tissue proteins in T-PER (Thermo Fisher Scientific) or HSB (50 mM HEPES, pH 7.4; 137 mM NaCl; 1% NP-40 (v/v); 2 mM EDTA; 1 mM PMSF; protease inhibitors cocktail (Roche Diagnostics, Meylan, France) lysis buffer. Homogenization was performed using first a Polytron TP-20 Homogenizer (Kinematica, Lucerne, Switzerland) and then a sonicator (3 x 10-seconds pulses at 80% magnitude). After two centrifugations at 13000 g for 45 min at 4°C to remove insoluble material from the supernatant, the protein concentration of every soluble extract was determined using the Bradford assay kit according to the manufacturer’s instructions (Thermo Fisher Scientific).

#### 9-Affinity determination evaluated by SPR

The interactions between the anti-αIIbβ3 TEG4-2c scFv and the integrin αIIbβ3 were analyzed by SPR sensing using BiacoreTM 3000 (GE Healthcare), according to the manufacturer’s instructions. HBS-EP buffer (0.01 M HEPES pH 7.4, 0.15 M NaCl, 3 mM EDTA, 0.005% v/v Surfactant P20) (GE healthcare life sciences, France) with 2 mM Ca^2+^ was used as a running buffer. The purified αIIbβ3 was first dialysed in the running buffer, diluted in 10 mM Na-Acetate, pH 5.5, then immobilized onto the sensor surface of a Biacore sensor chip CM5 to an immobilized ligand density (RU) of about 8000, using an amine coupling kit. Then, anti-αIIbβ3 TEG4-2c scFv diluted into the running buffer at concentrations ranging from 90 to 400 nM was allowed to flow through the sensor chip for 5 min at a flow rate of 20 μL/min and dissociation of bound analyte was allowed to proceed for 15 min before chip regeneration with NaOH 20mM.

#### 10-Affinity determination evaluated by BLI: Octet Red96

Octet instrument is a label-free detection system that exploits optical principle to read bimolecular interactions, the bio-layer interferometry (BLI). The interactions between the anti-αIIbβ3 TEG4-2c scFv and blood platelets were analyzed using Octet (Red96) in platelet buffer (NaCl 137 mM, KCl 2 mM, NaH_2_PO_4_ 0.3 mM, MgCl_2_ 1 mM, Glucose 5.5 mM, Hepes 5 mM, Bicarbonate-Na 12 mM pH6). The purified TEG4-2c scFv was loaded on a HIS2 biosensor (anti-penta Histidine Ab optical fiber based sensor) at 21 μg/mL. Platelets were diluted in PBS, coated into 96 well plate and analyzed at different concentrations: 5x10^5^ to 5x10^8^/mL, corresponding to integrin αIIbβ3 molarities from 42 pM to 42 nM. The plate is shaken during reading to create an “orbital flow”. Controls with no scFv and no platelets were performed to check for nonspecific binding and signal drift, respectively.

#### 11-Immunohistochemistry Analysis on Murine and Human Atherosclerotic Sections

All animal studies were approved under the N°50120192-A by the Animal Care and Use Committee of Bordeaux, France. The studies were performed in accordance with the ethical standards laid down in the declaration of Helsinki. ApoE^-/-^ mice were fed a high-cholesterol diet for 21 weeks to allow for the development of atherosclerotic lesions, afterward they were terminally anaesthetized using a lethal dose of isoflurane. The chest was opened and the aorta retrieved from the aortic arch to the iliac bifurcation. The lesions-rich areas were subsequently isolated, fixed and embedded in paraffin. New Zealand white rabbits were prepared as described in Methods, section 8.

Human coronary arteries were harvested from patients with end-stage heart failure having undergone heart transplantation. All of the clinical interventions took place at Haut-Lévèque Hospital (Pessac, France). All work with tissues from human subjects had been approved by the CPP committee (Comité de Protection des Personnes Sud-Ouest et Outre Mer) of Bordeaux and from the Research Ministry in France (Authorization number DC -2016- 2724). The CPP committee waived the need for patient written consent because surgical waste no longer attached to the person is considered "RES NULLIUS ". Patients were informed by the clinicians; if they did not express their opposition to research, the de-identified samples were immediately processed and embedded in paraffin.

Paraffin-embedded sections of arterial tissue from mouse, rabbit or human were used in IHC experiments. The following antibodies were tested on sections from each specie: TEG4-2c scFv (human fragment antibody, tested between 20 and 40 μg/ml), anti-CD41 (anti-αIIb rabbit antibody, working dilution 1:200, Abcam, France), AP2 (anti-αIIbβ3 mouse antibody, 10 μg/mL), RAM11 (mouse antibody, working dilution 1:50) and PGM1 (mouse antibody, working dilution 10ug/ml) anti-CD68 antibodies targeting rabbit and human macrophages respectively (Dako, Les Ulis, France).

Paraffin-embedded sections were deparaffinised, rehydrated, and heat mediated antigen retrieval was performed with Tris-EDTA pH 9 buffer following the specifications of Abcam, Paris, France (www.abcam.com/ps/pdf/protocols/ihc_p.pdf‎). Endogenous peroxidase was then blocked with 3% H_2_O_2_ in water, for 15 min. After washing in PBS 1X + 0.025% Triton (PBST), nonspecific binding was blocked with PBS 1X + 0.2% Triton + 2% bovine serum albumin (BSA) for 1 h at room temperature.

Afterwards, antibodies were applied overnight at 4°C, diluted at 20 μg/mL in PBS 1X + 1% BSA. The following day, three washes with PBST were performed. To detect TEG4-2c scFv fragments, an HRP-conjugated antibody specific to 6His (working dilutions 1:250) was applied to the sections for 1 h at room temperature. HRP-conjugated secondary antibodies specific to (1) rabbit IgG H+L (Beckman Coulter), (2) mouse IgG H+L (Beckman Coulter) were respectively applied for commercial primary (1) anti-CD41, (2) RAM-11, PGM1 and AP2 antibodies.

After a further three washes with PBST, staining was performed by adding the peroxidase substrate diaminobenzidine (DAB substrate kit, Eurobio/ABCys, Les Ulis, France) with H_2_O_2_. It yielded a yellow brown deposit within 10 min at room temperature. After a wash in dH_2_O to stop the enzymatic reaction, slides were counterstained in hematoxylin, dehydrated and mounted.

## Results

### 1-Monitoring TEG4-2c scFv large scale production in *Pichia pastoris*

After the transformation of *P*. *pastoris* with linear pPICZαA-TEG4-2c plasmid, up to 60 clones were selected on 2 mg/mL Zeocin agar plates. The colony PCR analysis confirmed the presence of TEG4-2c scFv gene in 16 tested clones. Six clones were evaluated for their capacity to produce and secrete TEG4-2c scFv in a shake flask. Finally, the clone TEG4-2c A2 was selected for further analysis and the production scaled up in a 5 L benchtop bioreactor.

**Fig 2:**
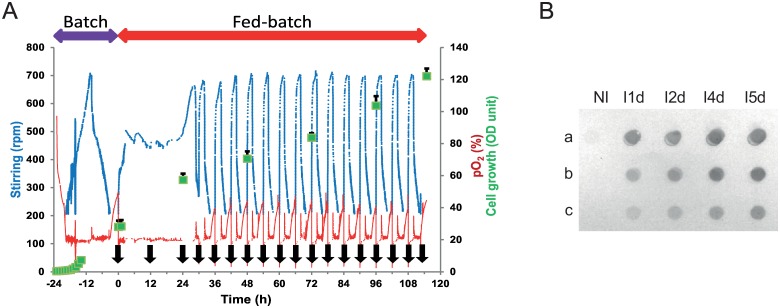
TEG4-2c scFv production process. A: Fed batch fermentation history plot. Stirring, pO_2_ and OD_600_ values are plotted versus time during the cultivation of *P*. *pastoris* in BMGY medium. Cultures were induced with methanol at t = 0 (24 h after starting the batch phase) during the fed batch phase the methanol was added every 12 h or 6h (black arrows) to a final concentration of 0.6%. The average values are shown with error bars representing the standard deviation (n = 5). 1 OD_600_ unit was equivalent to 0.29 mg/mL dry weight. **B: Dot-blot analysis of supernatants from recombinant *P*. *pastoris* culture**. Fifty microliters samples from non-induced culture (NI) and from day 1 to day 5 induced cultures (I1d to I5d) were undiluted (a) or diluted (b = 1:10; c = 1:50) and blotted on a nitrocellulose membrane. The recombinant TEG4-2c scFv were detected with the Anti-6His antibody and revealed by colorimetric analysis.

Five culture batches were carried out on a 5 L benchtop bioreactor and exhibited a similar growth profile (data not shown). The culture conditions were maintained as described in the experimental section. The dissolved oxygen level was maintained throughout the production batch at 20% by a combination of agitation and aeration systems, in a cascading manner to improve the oxygen supply during the growth of cells (Fig 2A). The yeast growth was exponential during the batch culture with glycerol as the substrate; the glycerol was exhausted after 20 to 24 h culture and the cell density reached 28 OD units corresponding to 8.1 g/L (dry weight).

A fed-batch phase was initiated by adding methanol every 12 h to induce the scFv production. To optimize the efficiency of scFv production during the 4 last days, the methanol feed frequency was every 6 h. Following each substrate addition event, there was a sharp increase in agitation indicating the active consumption of substrate and sustainable growth of cells. Stirring, which again diminished upon substrate consumption, was raised following the consecutive methanol injection. During the fed-batch phase, a linear increase in yeast biomass was observed 24 h after induction. A growth rate of 0.23 g L^-1^h^-1^ was maintained during 5 days; the cell density was also increased by a 4.3 fold and reached 35 g/L (dry weight).

Dot-blot analysis was performed to evaluate the TEG4-2c scFv expression each day after induction in the culture broth. Recombinant TEG4-2c was only produced upon induction of transformed X-33 cells with methanol. This was clearly confirmed by the absence of signal into the samples before methanol feeding and into the samples from X33 *Pichia* cells transfected with the empty pPICZαA plasmid. The anti-αIIbβ3 TEG4-2c scFv was expressed from the first day in a soluble form into the cells culture medium (Fig 2B).

### 2-Purification of TEG4-2c scFv by IMAC

**Fig 3:**
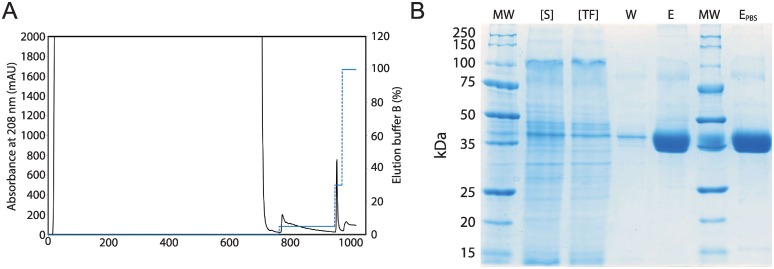
**Purification of recombinant TEG4-2c scFv A: IMAC chromatogram**. The HisTrap Excel resin (5 mL) was equilibrated with 50 mM Tris-HCl pH 7.5, 500 mM NaCl (buffer A at a flow rate of 3 mL/min). *Pichia pastoris* expression broth supernatant containing the TEG4-2c scFv was injected into the column. The column was then washed with buffer A until absorbance at 280nm reached the baseline. (Dot Line): The elution was carried out in two steps using 5% and 30% buffer B (50 mM Tris-HCl pH 7.5, 500 mM NaCl, 500 mM imidazole) corresponding respectively to 25 mM and 150 mM imidazole. **B: Electrophoretic analysis of one step IMAC purification of recombinant TEG4-2c scFv**. 12% SDS-PAGE stained with colloidal blue, MW: molecular weight ladder (KDa). [S]: 5x concentrated culture supernatant. [TF]: 5x concentrated flow-through. W: 25 mM imidazole washing fraction. E: 150 mM elution fraction. E_PBS_: Elution fraction dialyzed against PBS.

For the purification of recombinant TEG4-2c scFv, optimal performance was obtained when an intermediate washing step with 25 mM imidazole was used to remove the contaminating proteins weakly bound to the column, after which recombinant TEG4-2c scFv was eluted using 150 mM imidazole (Fig 3A).

Typically, 30 mg of TEG4-2c scFv were produced and secreted by *P*. *pastoris* into 1L broth medium after 5 days of growth. The scFv content into this supernatant was estimated to 1.4% of the total proteins. After the IMAC purification, the yield was around 22 mg TEG4-2c scFv from 1L culture medium. This represents a recovery of 70% of produced scFv with a high purity (> to 80%). This one step chromatography led to a 57 fold purer product with highly concentrated solution of TEG4-2c scFv (Table 1).

**Table 1:** IMAC Purification of scFv TEG4-2c produced and secreted by *P*. *pastoris*. Data are standardized for 1 L culture media; the values are the mean of 7 independent experiments ± SD values.

	Protein^a^ (mg/mL)	scFv^b^ (μg/mL)	Volume (mL)	Total protein^a^ (mg)	Total scFv^a^^b^ (mg)	Step recovery (%)	Step purification (fold)
**Supernatant**	2.190 ± 0.367	30.7 ± 4.3	1000	2190 ± 367	30.7 ± 4.3	100	0
**Flow through**	1.983 ± 0.377	ND	1000	1983 ± 377	ND	ND	ND
**Wash step**	0.167 ± 0.036	16.7 ± 3.3	75 ± 5	12.53 ± 1.64	1.253 ± 0.25	ND	ND
**Elution step**	0.866 ± 0.136	695 ± 35	31 ± 2	26.85 ± 1.52	21.56 ± 3.01	70.3 ± 0.6	57.35

^a^ Protein concentration was determined by BCA protein assay, using bovine serum albumin as standard.

^b,c^TEG4-2c scFv into the supernatant and the purity was estimated by densitometric quantification of corresponding lane of SDS-PAGE 12% acrylamide gel stained by colloidal blue, thanks to Bio-Rad Image Lab^™^.

ND: not determined.

The eluted fractions were analyzed by SDS-PAGE (Fig 3B), which revealed a major band of 35 kDa from the elution step fractions, corresponding to the expected molecular mass of recombinant TEG4-2c scFv. A weaker band of 75 kDa was also present in this lane, but the mass spectroscopy analysis revealed that TEG4-2c scFv was the major protein present in this fraction (data not shown). This data strongly suggests that the higher molecular weight protein was indeed TEG4-2c scFv dimer.

The elution fraction was dialyzed against PBS buffer. The SDS-PAGE (Fig 3B-E_PBS_) shows the purified TEG4-2c scFv as a single thick band with an estimated purity higher than 80%. The profile of TEG4-2c scFv into PBS buffer (used for ELISA and affinity measurement) is similar to the elution fraction (E) obtained at the end of IMAC. The final overall yield of TEG4-2c was 0.7 mg/mL; it represents 70% of initial product. However, upon SDS-PAGE analysis, it was observed that some amount of our protein of interest was also lost in the flowthrough and washing steps.

### 3-Analysis of TEG4-2c scFv binding to platelets by flow cytometry and ELISA tests

#### 3.1-Flow cytometry analysis using TEG4-2c scFv

**Fig 4:**
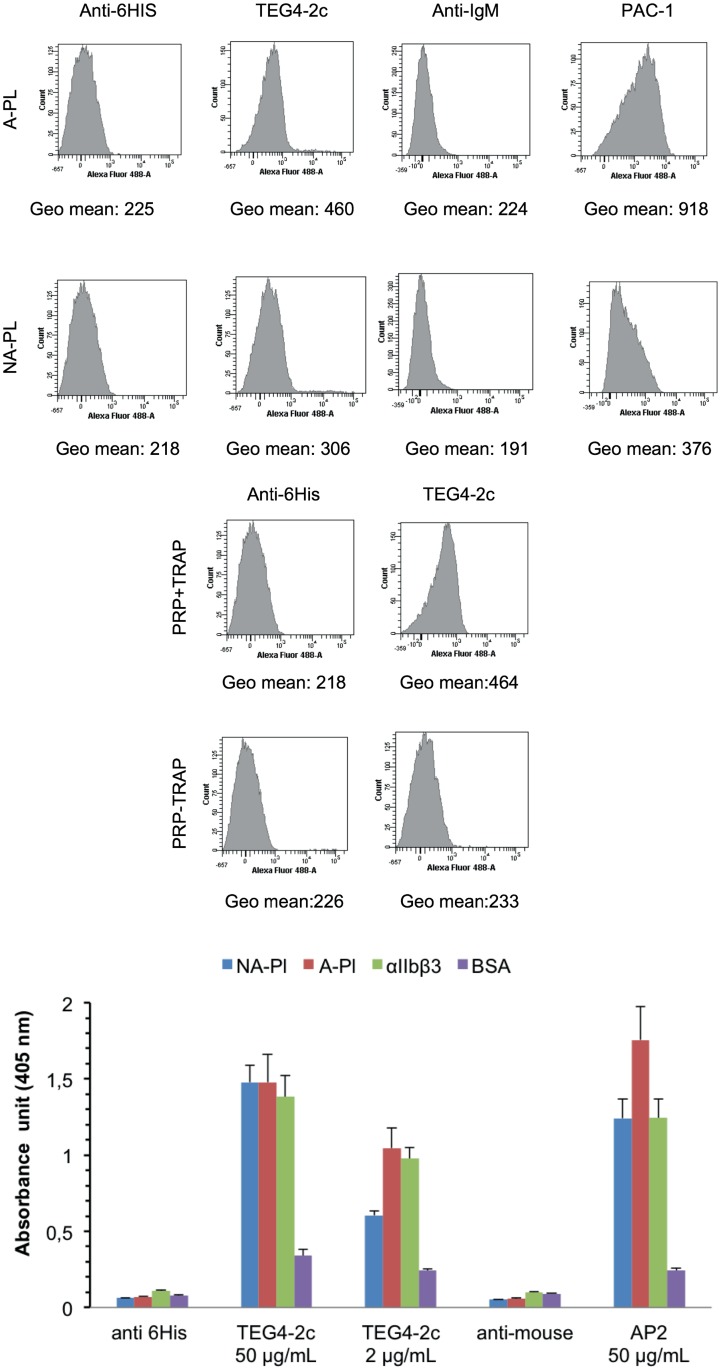
Binding assessment of TEG4-2c scFv to human platelets by flow cytometry and ELISA tests. **A**: Binding of TEG4-2c scFv on thrombin-activated human (A-PL) or non-activated—platelets (NA-PL) analysed by flow cytometry. PAC-1 IgM murine antibody serves as a positive control. Binding of antibody to the platelets was further detected by incubation with Alexa Fluor 488 anti-6His or anti-mouse IgM antibodies. Negative controls were secondary antibody only. Histograms depict representative data ± SD of three independent experiments. Quantitative fluorescence intensities (in Geo mean) are stated under each respective histogram. **B**: Binding of TEG4-2c scFv on TRAP-activated-human (+ TRAP) or non-activated platelets (-TRAP) analysed by flow cytometry. Quantitative fluorescence intensities (in Geo mean) are stated under each respective histogram. **C**: Representative whole cell (A-PL, NA-PL) and purified proteins (αIIbβ3, BSA) ELISA with TEG4-2c scFv. A murine anti-αIIbβ3 antibody AP2 was used as positive control. Negative controls were secondary antibody only. Binding of antibodies was visualized via HRP-6His or HRP-anti-mouse IgG. OD value represents absorbance at 450 nm. Plots represent the mean values ± SD (n = 3)

Flow cytometry analysis showed a better recognition of washed activated platelets versus non-activated ones (Fig 4A). PAC-1, an IgM murine antibody specific to the activated conformation of αIIbβ3 was included as a positive control of the experiment. The slight recognition of resting platelets might be due to their activation during the purification process. Activation of platelets is a problem classically encountered during their handling and processing. To minimize manipulation steps, the binding specificity of TEG4-2c scFv was checked on platelets within plasma (analysis on PRP). The results (Fig 4B) highlighted a binding specificity for platelets activated in PRP with TRAP peptide whereas resting platelets were not recognized.

#### 3.2-ELISA tests

The reactivity of TEG4-2c scFv produced in *Pichia pastoris* was then measured on human thrombin-activated and non-activated platelets and on purified αIIbβ3 by ELISA (Fig 4C). We have to underline that, here again, the coating of platelets on ELISA wells may itself induce their activation. However, a better recognition of activated platelets is reported with TEG4-2c scFv, especially when using lower concentrations. AP2, a murine antibody directed against αIIbβ3 was included as a positive control of the experiment.

### 4-Binding of scFv TEG4-2c to αIIbβ3 investigated by SPR and BLI

Affinity of TEG4-2c scFv was determined either by surface plasmon resonance (SPR) with a BIACORE device on purified αIIbβ3 or using the Bio-Layer Interferometry (BLI) technology with an OCTET instrument on lyophilized platelets resuspended from a freeze-dried solution.

#### 4.1-BIACORE analysis

**Fig 5:**
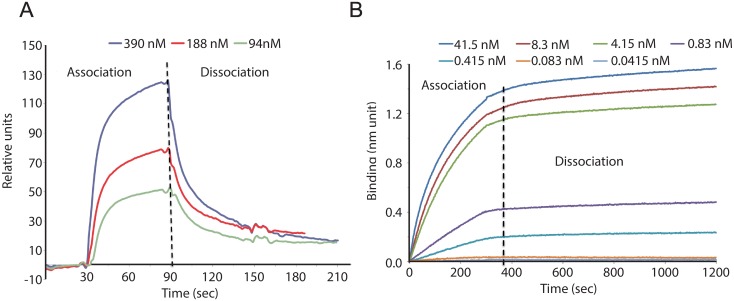
Binding of scFv TEG4-2c to αIIbβ3 by SPR and to whole platelets by BLI. **A: SPR sensorgrams**. The ligand αIIbβ3 was immobilized on CM5 chip by amine coupling with a density of 8000 RU. Serial dilutions of TEG4-2c in HBS-EP running buffer were injected over the ligand corresponding to 94, 188 and 390 nM. Sensorgrams show a binding concentration-dependent of TEG4-2c scFv. **B: BLI analysis**. TEG4-2c scFv (ligand) was loaded on HIS2 biosensor (anti-penta Histidine Ab) at 21 μg/mL. Whole platelets (analyte) concentrations converted into αIIbβ3 molarities were: 41.5, 8.3, 4.15, 0.83, 0.415, 0.083 and 0.0415 nM. Additionally one sensor pair was used to record the buffer reference signals. TEG4-2c scFv reacts with αIIbβ3 in its natural conformation in a concentration dependent manner.

In this first experimental session using SPR technology, TEG4-2c scFv was injected at 3, 6 and 12,5 μg/ml on immobilized αIIbβ3, corresponding respectively to 94, 188 and 390 nM. The binding was concentration-dependent (Fig 5A) but the three sensorgrams could not be fitted using the simple Langmuir model, likely because of the presence of a more complex interaction, implying monomers and dimers in the purified fractions.

#### 4.2-Binding by BLI

This second set of experiments performed using the Octet instrument aimed at determining the interactions between TEG4-2c scFv and blood platelets (Fig 5B). This is more informative because this strategy allows evaluating the binding on αIIbβ3 in its natural conformation. TEG4-2c scFv immobilized through an anti-6His coating on an optical fiber based sensor was immersed in a solution of platelets (seven different concentrations were used) contained in the well of a 96- well plate. The plate is shaken during reading to create an "orbital flow". The different platelet concentrations, from 5x10^5^ to 5x10^8^/ml, were converted into integrin αIIbβ3 molarities by taking into account the number of αIIbβ3 per platelet (50 000). Controls with no scFv allowed checking for non-specific binding of the platelets on the sensors. These controls were subtracted from the curves obtained with TEG4-2c scFv. Other controls were also performed with no platelets (only buffer), assessing the signal drift due to the potential release of scFv from sensors.

Octet experiments showed good interactions between TEG4-2c scFv and platelets at nanomolar concentrations superior to 0.4 nM. No Kd could be calculated because no dissociation was observed. However, considering the binding at 0.8 and 4 nM, the affinity should be in the nanomolar range.

### 5-Evaluation of atheroma burden recognition by IHC and ELISA tests

**Fig 6:**
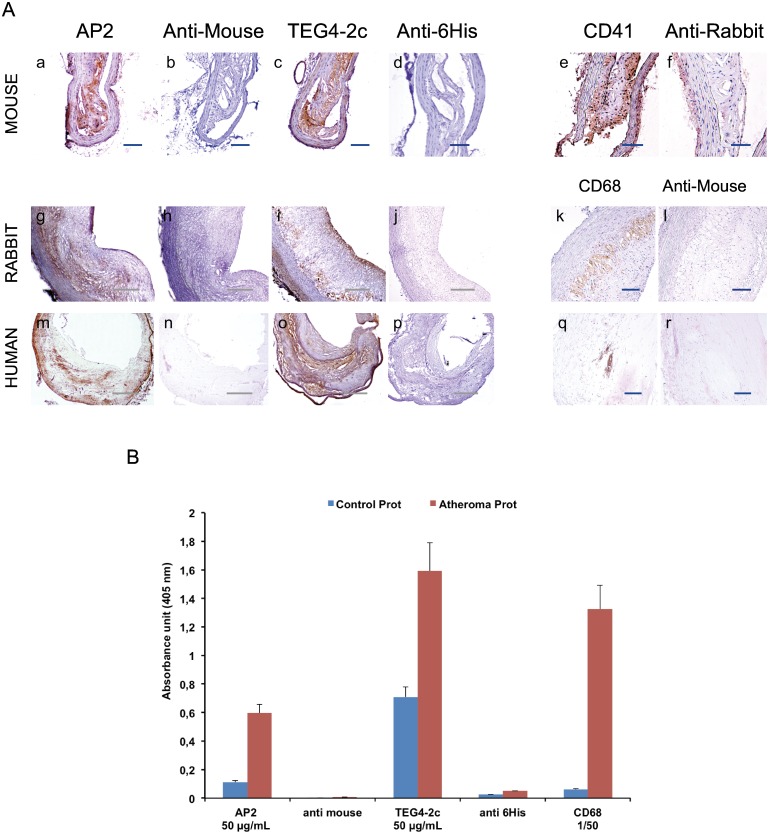
Comparison of the immunoreactivity of TEG4-2c scFv to atherosclerotic tissues of different species by IHC analysis and ELISA assays. **A** (**a-r)**: IHC assays on atherosclerotic tissues: similarly to positive controls e.g; anti-CD41 (anti-αIIb) (e), RAM11 and PGM1 (anti-CD68 antibodies targeting rabbit and human macrophages respectively) (k, q) and AP2 (anti-αIIbβ3 antibody) (a; g; m), TEG4-2c scFv specifically recognizes the injured areas of the aorta sections from different species (c; i; o). Binding of antibodies was visualized via HRP-anti-6His (scFv); HRP anti-rabbit IgG (CD41) and HRP anti-mouse IgG (RAM11, AP2). Negative controls were secondary antibody only (b; d; f; h; j; l; n; p; r). Nuclei were counterstained with hematoxylin **B**: ELISA tests on atheromatous and healthy aorta proteins: TEG4-2c shows a better recognition of atheromatous proteins. RAM11 and AP2 were used as positive controls. Negative controls were secondary antibody only. Binding of antibodies was visualized via HRP-6His or HRP-anti-mouse IgG. OD value represents absorbance at 450 nm. Values represent mean (n = 3) ± SD (error bars materialized the SD)

As the final aim of the project is to target platelets colonizing the plaque, we evaluated the ability of TEG4-2c scFv to recognize platelets within aorta lesional sections of animal models of atherosclerosis as well as aorta sections recovered from human coronary specimens (Fig 6A). The reactivity was also tested on proteins isolated from the extracted aorta from atheromatous and healthy rabbits by ELISA (Fig 6B).

#### 5.1-IHC

The immunohistochemistry data were in accordance with ELISA with a huge recognition of platelets in all analyzed sections (Fig 6A). An antibody directed against murine αIIb (anti-CD41) and antibodies directed against rabbit and human macrophages (anti-CD68) over-expressed in the atheroma burden were included as positive controls. We also compared the targeting of TEG4-2c scFv with that of AP2, an antibody we have already demonstrated able to label mouse, rabbit and human platelets.

#### 5.2-ELISA tests

ELISA assay clearly showed a better recognition of atheromatous proteins with TEG4-2c scFv and AP2 antibodies (Fig 6B). An anti-CD68 macrophage rabbit antibody was included as positive control. We confirmed by mass spectroscopy analysis (data not shown) that the αIIbβ3 integrin is also present in healthy aorta but to a lesser extent.

## Discussion & Conclusion

In the present study, TEG4-2c scFv was expressed at high-level in *Pichia pastoris* using a fed-batch fermentation system monitored by pO_2_ level. We produced the TEG4 scFv with cysteine tags at the end of the C-terminal sequence for site-specific conjugation to contrast agents, precluding the loss of reactivity potentially occurring when the grafting process affects antigen-recognition sites. TEG4 scFv had been previously expressed in *E coli*.[27] Unfortunately, despite optimization tests leading to high yields of cytoplasmic production, proteins also frequently accumulated into inclusion bodies (data not shown). In bacterial systems, many scFv can be produced into the periplasmic space but they are obtained with a very low yield. Higher levels of production can be achieved in inclusion bodies, with the limitation of the presence of insoluble scFv aggregates and the need for subsequent *in vitro* folding that make the use of this bacterial system not attractive for the large scale production of scFv. In addition, many authors have described that the final yield of scFv was only a small percentage of produced proteins with a low specificity for targets.[33,34]

So we chose to use the *Pichia* yeast as an alternative expression system. Indeed, *Pichia pastoris* is an attractive system for low cost-effective large-scale production of heterologous proteins. This type of production, characterized by the secretion of the protein of interest into the culture medium, can be easily scaled up and reach a GRAS (Generally Recognized As Safe) status. The concentration of highly pure TEG4-2c scFv obtained after one chromatography step (IMAC) was up to 600 μg/mL. A production of 30 mg scFv per liter of culture was achieved. This value was lower than that obtained for an scFv anti-carcinoembryonic antigen,[33] but in accordance with the yields obtained for the majority of Fab and antibody fragments expressed in *P*. *pastoris* [35–37] and sufficient for grafting purposes.

This new scFv format with cysteines included for grafting purposes was tested by ELISA, cytometry and IHC and all the experiments concurred to a specific recognition of platelets, from human or animal model origin and of atheroma issuing from coronary biopsies or animal lesional tissues. Bio-layer interferometry was used for evaluating the affinity of TEG4-2c scFv against platelets because this approach is more relevant than SPR analysis on purified antigen to mimic the *in vivo* behavior. No real Kd value could be extracted from the curves because of an absence of dissociation. This could be explained by the rebinding of the same platelet on the immobilized ligand because of the presence of 50 000 αIIbβ3 per platelet. Indeed, when the platelet is captured on the surface, one αIIbβ3 can dissociate while another re-binds on the scFv. As a consequence the off-rate is slower than it would be by using isolated αIIbβ3. Nevertheless, the bio-layer interferometry underlines a good affinity of TEG4-2c scFv for platelets with recognition in the nanomolar range. Moreover, the flow cytometry results with PRP are in favor of a preferential recognition of activated platelets. This approach, compared to ELISA assays or cytometry performed on washed platelets is the only one that allows recognition in physiological conditions.

The rationale for targeting activated platelets is that they are highly trapped within atherosclerotic lesions not only in thrombi and intraplaque hemorrhage but also in the atheroma burden, around necrotic areas and neovessels, mainly because of the presence of leaky vessels, blood extravasation and haemorrhage.[18] Preferential recognition of activated platelets is to be considered to avoid *in vivo* elimination of the probe by circulating resting platelets.

An important point to be underlined is that scFv TEG4-2c recognizes human platelets as well as murine or rabbit platelets. The use of a human antibody able to target epitopes on the integrin shared by animal models and humans is of particular interest for pre-clinical studies in animal models of the disease and for direct transfer into the clinic. The use of an antibody of human origin is appealing, considering the need for repeated injections in humans to follow the progression of the pathology. Indeed, the human scFv should limit the induction of anti-antibodies in patients as compared with murine origin; it also avoids any additional humanization step that could impair the reactivity. This antibody equipped with cysteine tags is now ready to be used to functionalize contrast agents for MRI to serve as imaging agents for atherosclerosis.

In conclusion, even if the production of antibody fragments by *P*. *pastoris* is not always a success story,[38] our findings demonstrated that *P*. *pastoris* provided TEG4-2c scFv at a high concentration without aggregates and claiming a good purity. Its functionality against activated platelets and atheroma tissues was proven, paving the way for the success of grafting tests. Our results definitely point out the *P*. *pastoris* expression system as an adapted tool to produce scFv for use in diagnostic or therapeutic applications as well.

## Abbreviations

A-PL: Activated Platelets
BLI: Bio-Layer Interferometry
ELISA: Enzyme-Linked Immunosorbent Assay
HuAb: human antibody
IHC: Immunohistochemistry
IMAC: Immobilized Metal Affinity Chromatography
NA-PL: Non Activated Platelets
OD: Optical Density
PRP: Platelet Rich Plasma
RU: Relative Units
scFv: single-chain variable fragment
SPR: Surface Plasmon Resonance
VH: Heavy Chain
VL: Light Chain
